# Transcriptome changes between compatible and incompatible graft combination of *Litchi chinensis* by digital gene expression profile

**DOI:** 10.1038/s41598-017-04328-x

**Published:** 2017-06-21

**Authors:** Zhe Chen, Jietang Zhao, Fuchu Hu, Yonghua Qin, Xianghe Wang, Guibing Hu

**Affiliations:** 1 State Key Laboratory for Conservation and Utilization of Subtropical Agro-bioresources, South China Agricultural University, Guangzhou, China; 2 Key Laboratory of Biology and Genetic Improvement of Horticultural Crops (South China), Ministry of Agriculture, College of Horticulture, South China Agricultural University, Guangzhou, China; 3 Institute of Tropical Fruit Trees, Hainan Academy of Agricultural Science, Haikou, China

## Abstract

Plant grafting has been practiced widely in horticulture and proved as a useful tool in science. However, the mechanisms of graft healing or graft incompatibility remain poorly understood. In this study, *Litchi chinensis* cv. ‘Jingganghongnuo’ homograft (‘J/J’) and ‘Jingganghongnuo’/‘zhuangyuanhong’ heterograft (‘J/Z’) as compatible and incompatible combination, respectively, was used to study transcriptional changes between incompatible and compatible graft during graft union formation. Anatomical observation indicated that three stages (2 h, 14 d and 21 d after grafting) were critical for graft union formation and selected for high-throughput sequencing. Results indicated 6060 DEGs were differentially expressed in the compatible combination and 5267 DEGs exhibiting in the incompatible one. KEGG pathway enrichment analysis revealed that DEGs were involved in metabolism, wound response, phenylpropanoid biosynthesis and plant hormone signal transduction. The expression of 9 DEGs annotated in auxin pathway was up-regulated in compatible combination than that in incompatible combination. The IAA concentration confirmed that the IAA might promote the graft compatibility. In addition, 13 DEGs related to lignin biosynthesis were differentially expressed during graft healing process. Overall, our results provide abundant sequence resources for studying mechanisms underlying graft compatibility and establish a platform for further studies of litchi and other evergreen fruit trees.

## Introduction

Grafting is an ancient asexual plant propagation technique that has been widely practiced in agriculture and horticulture. Fruit trees are grafted for propagation, avoiding a juvenile state, modifying plant growth or increasing stress resistance^1^. An example occurred in the litchi industry. In China, top-grafting generally aims at modifying the litchi variety of a mature tree. Top-grafting litchi trees begin producing fruits in 1–2 years, greatly reducing the juvenile period. In addition, plant grafting has been used as a convenient tool to study the long-distance movement of proteins, hormones and RNAs^1–3^. Despite the widespread use in horticulture and science, the mechanism of plant grafting remains poorly understood^3^.

In grafting, the shoot of one plant, termed the scion, is grafted onto the root of a different plant, termed the rootstock. Successful grafting requires the rejoining of a functional vascular system between the scion and rootstock, which is a complex biochemical and structural process^4^. Several reports have described the key events for graft healing at the graft junction with regard to the histological and physiological aspects^5–7^. After grafting, ruptured cells collapse and adhere to the opposing tissue at the graft junction, where a mass of pluripotent cells termed callus is formed. The callus cells differentiate into vascular tissue-phloem and xylem to reconnect the scion and rootstock across the graft junction^3, 6^. However, successful grafting termed ‘graft compatibility’ only occurs among the scion and rootstock with close taxonomic affinity. Incompatible grafts fail to form a vascular union between the scion and rootstock duo to insufficiently close genetic relationship. The reasons for graft incompatibility are as yet insufficiently understood^2^.

At the molecular level, the mechanism of graft healing process remains unclear, and no genes have been identified to be critical for graft union formation^1^. As a wound healing process for plants, graft union formation presumably requires considerable re-programming of gene expression, protein translation, and metabolism^8^. Zheng *et al*.^9^ used cDNA-AFLP to examine the gene expression in hickory (*Caryaca thayensis*) during graft healing process, and the results indicated that the genes involved in indole-3-acetic acid (IAA) transport, cell cycle, signal transduction, and metabolism were differentially expressed. Recently, graft union development and vascular reconnection in model plant *Arabidopsis* has been studied at the histological and transcriptional level^3, 10^. Microarray data revealed that graft union development was shown to involve wound and hormone signaling^10^. Melnyk *et al*.^3^ identified the genes involved in vascular reconnection at the graft junction and revealed that *ALF4* and *AXR1* were required locally below the graft junction to promote graft formation. However, grafting in woody fruit trees seem to be different from hypocotyl grafting^11^. Cookson *et al*.^8^ investigated the transcriptome of grapevine rootstock and graft interface tissues sampled 3 d and 28 d after grafting of over-wintering stems in the spring, results indicating that many differentially expressed genes were related to the activation of stem growth and metabolic activity in the spring. More recently, proteomic-based study was performed in watermelon to reveal the proteins involved in vascular connections between the rootstock and scion^12^.

Litchi (*Litchi chinensis* Sonn.) is one of the most important tropical and subtropical fruit trees in the Sapindaceae family. Grafting has been practiced in litchi for several hundred years in China. However, grafting success is variable because of rootstock-scion incompatibility. In a previous study, our group has shown that there were significant differences on the survival rate of ‘Jingganghongnuo’ grafted onto different litchi cultivars as rootstocks^13^. The compatible combinations had higher SOD, POD and PPO activities than incompatible combinations^13^. However, to the best of our knowledge, the underlying causes of graft incompatibility in litchi are unknown.

It is possible that the differentially expressed genes between compatible and incompatible graft combination revealed at transcriptome level could provide key information about the genes related to the vascular connections between the rootstock and scion. Recent years, an increasing number of studies have confirmed that the next generation sequencing is well-suited for studying transcriptome profiles in fruits^14–16^. To date, transcriptomic studies in litchi have been primarily focused on shading^17^, response to reactive oxygen species^18^, floral initiation^19^, and maturation^20^. Digital gene expression is a tag-based transcriptome sequencing method in which the gene expression level is measured by counting the number of individual mRNA molecules produced from each gene, which enables the digital gene expression protocol more suitable and affordable for comparative gene expression studies^21^. In this study, six digital gene expression libraries were constructed at different graft developmental stages and gene expression profiles were analyzed. By comparing anatomical and transcriptional differences in compatible and incompatible grafts at three different developmental stages, we hypothesize that genes related to wound response, IAA and signal transduction pathway might play a key role in litchi grafting healing process. This study would provide useful information for revealing the mechanism of graft healing in plant.

## Results

### Anatomical observation

**Figure 1:**
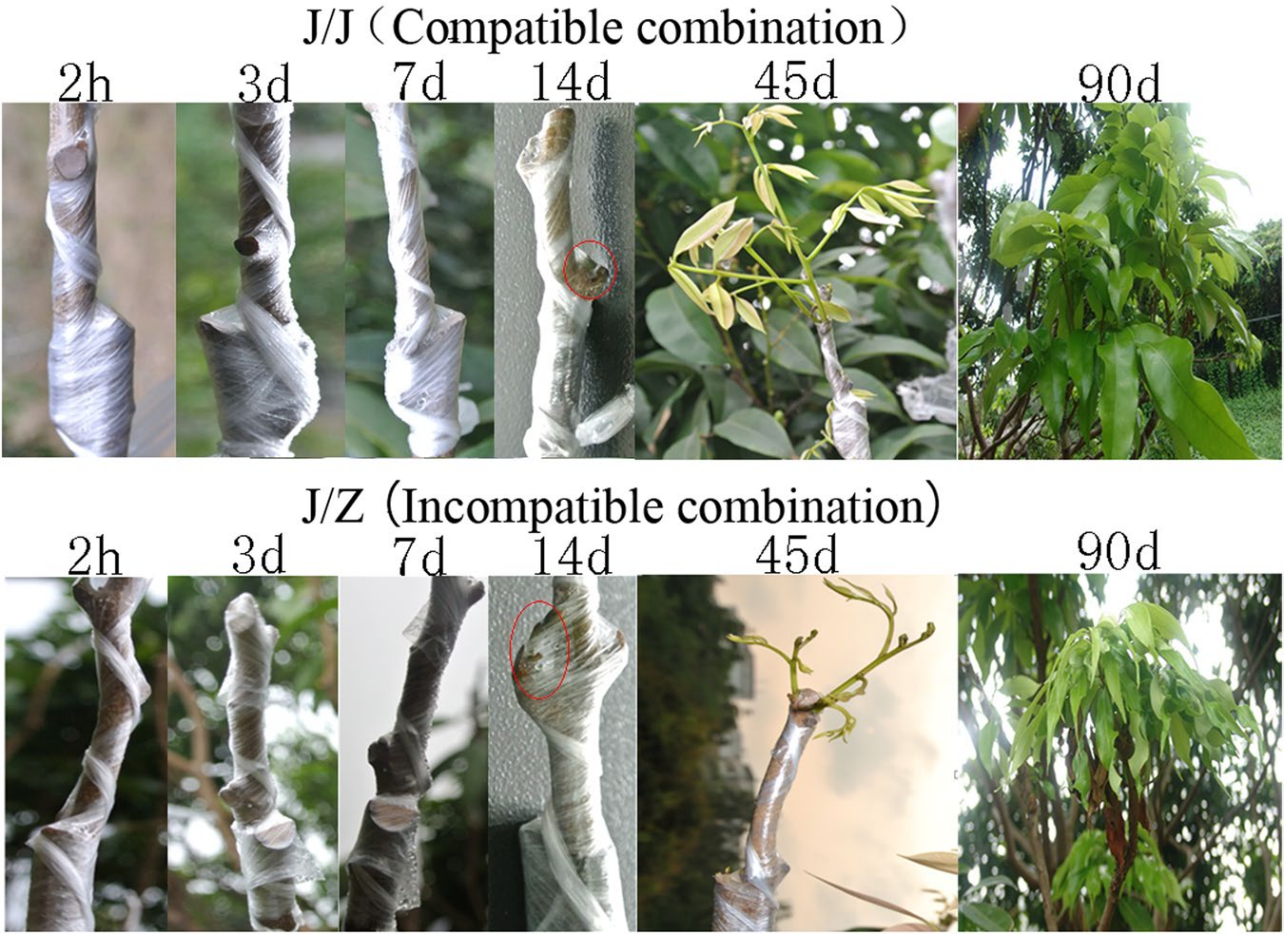
Field performance of the litchi compatible graft J/J and incompatible graft J/Z at 2 h, 3 d, 7 d, 14 d, 45 d and 90 d after grafting.

In our previous study, we found that Jingganghongnuo/zhuangyuanhong (‘J/Z’) litchi heterograft was incompatible and Jingganghongnuo litchi homograft (‘J/J’) was compatible^13^. Compared with ‘J/Z’ graft combination, ‘J/J’ graft combination had higher survival rate as shown in Figs 1 and S1, which is consistent with our previous results^13^. Therefore, ‘J/Z’ and ‘J/J’ graft combinations were selected to determine the transcriptional changes between incompatible and compatible graft combination in the present study.

**Figure 2:**
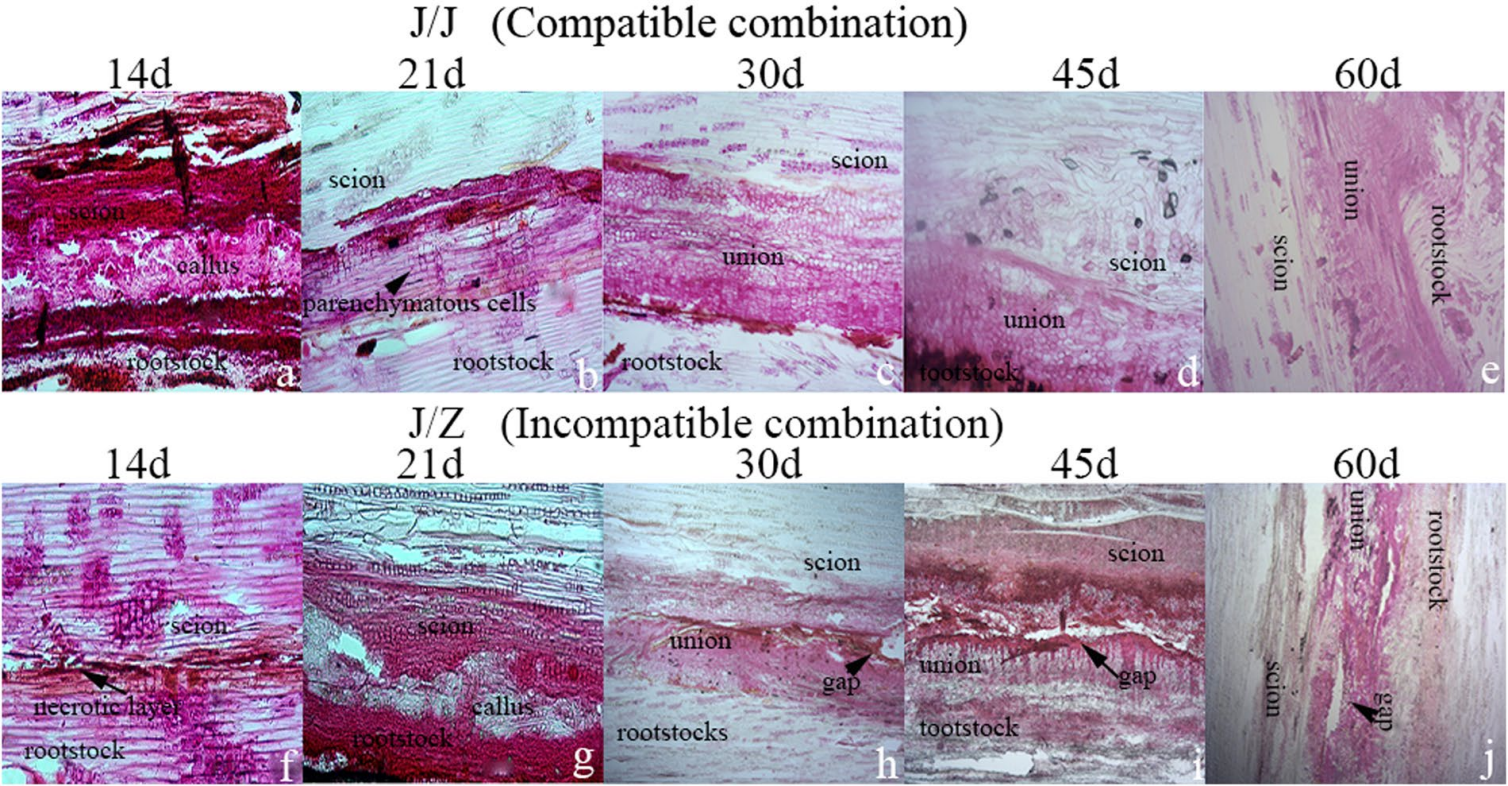
Anatomical analysis of the litchi compatible graft J/J (**a**–**e**) and incompatible graft J/Z (**f**–**j**) at 14 d, 21 d, 30 d, 45 d and 60 d after grafting.

To determine the vital stages of the graft union formation in litchi, the field performance of the graft compatibility and incompatibility was recorded at different stages after grafting (Fig. 1). The scion began sprouting 14 d after grafting in litchi (Fig. 1). According to the histological studies, there were huge differences between compatible and incompatible combination (Fig. 2). In the homograft J/J, callus filled up the spaces between the scion and rootstock interface holding them together tightly (Fig. 2a) 14 d after grafting. However, there still was necrotic layer at the graft interface in the heterograft J/Z (Fig. 2f). The parenchymatous cells close to the graft interface proliferated and adhered to the opposing tissue (Fig. 2b). The contact strengthened with time as the cells interdigitated, and connected the rootstock and scion together (Fig. 2c). The stem cell-like tissue differentiated and gave rise to new vascular tissues, which connected the xylem and phloem between the scion and rootstock (Fig. 2d,e). On the contrary, the heterograft J/Z showed a slower cambial initiation rate (Fig. 2g,h) and vascular complexes were abnormal (Fig. 2i,j) compared with the homograft J/J. Based on the results obtained from the field performance and anatomical observation, the compatible graft showed higher survival rate and had faster healing process than that of the incompatible one. Therefore, the gene expression differences of the graft interface of compatible homograft and incompatible heterograft were analyzed at 2 h, 14 d and 21 d after grafting using high-throughput sequencing.

### Digital gene expression library sequencing

Six libraries were constructed and sequenced using an Illumina genome analyzer. In total, 83,382,923 sequence reads, each 49 bp in length, were generated, encompassing 3.8 Gb of sequence data (Table 1). Each library was represented by at least 11 million reads, a tag density sufficient for quantitative analysis of gene expression^22^. The reads were mapped to the reference sequence. Results indicated that there were at least 58% total mapped reads for each samples (Table 1).

**Table 1:** RNA-seq reads in six RNA-seq libraries.

Sample ID	Total Reads	Total Base Pairs	Total Mapped Reads	Unique Match	Multi-position Match	Total Unmapped Reads
J/J 2 h	11748762 (100.00%)	575689338 (100.00%)	6922403 (58.92%)	5751212 (48.95%)	1171191 (9.97%)	4826359 (41.08%)
J/J 14d	11783646 (100.00%)	577398654 (100.00%)	7002348 (59.42%)	5777537 (49.03%)	1224811 (10.39%)	4781298 (40.58%)
J/J 21d	12050581 (100.00%)	590478469 (100.00%)	7087563 (58.82%)	5838291 (48.45%)	1249272 (10.37%)	4963018 (41.18%)
J/Z 2 h	12174395 (100.00%)	596545355 (100.00%)	7481779 (61.46%)	6207854 (50.99%)	1273925 (10.46%)	4692616 (38.54%)
J/Z 14d	11709807 (100.00%)	573780543 (100.00%)	7082819 (60.49%)	5846953 (49.93%)	1235866 (10.55%)	4626988 (39.51%)
J/Z 21d	11783924 (100.00%)	577412276 (100.00%)	6928775 (58.80%)	5722216 (48.56%)	1206559 (10.24%)	4855149 (41.20%)

### Functional categorization

In this study, a rigorous algorithm was used to identify differentially expressed genes (DEGs) in the six samples based on the method described by Audic & Claverie^23^. The genes with significantly different expression patterns were subjected to GO analysis. The results showed that most DEGs were classified into 3 functional categories: ‘biological process’, ‘cellular component’, and ‘molecular function’ (Figure S2). In the GO category of ‘biological process’, ‘metabolic process’ and ‘cellular process’ were the most highly enriched terms. The ‘cellular component’ related to ‘cell’, ‘cell part’ and ‘organelle’ was enriched. In the GO category of ‘molecular function’, ‘catalytic activity’, ‘binding’ and ‘transporter activity’ was also enriched. There was no difference between compatible combination and incompatible combination based on the GO categorization. However, the number of DEGs fell into each functional category was different. For example, there were 1946, 305, and 52 DEGs divided into ‘response to stimulus’, ‘immune system process’, and ‘antioxidant activity’ in compatible combination, respectively, meanwhile the number in incompatible combination was only 1442, 207, and 46, respectively. The results indicated that more DEGs related to stress induction participated in the graft union formation in compatible combination than incompatible combination.

The DEGs were also subjected to KEGG pathway enrichment analysis. 4244 DEGs could be annotated in the compatible combination, while 3209 DEGs could be annotated in the incompatible combination. Genes involved in metabolic pathways, biosynthesis of secondary metabolites, plant-pathogen interaction and plant hormone signal transduction were differentially expressed during graft union formation (Figure S3). It is worth noting that phenylpropanoid biosynthesis pathway was higher enriched in compatible combination (164 DEGs) than that in incompatible combination (120 DEGs).

### Identification of genes showing differential expression between graft compatibility and incompatibility

**Figure 3:**
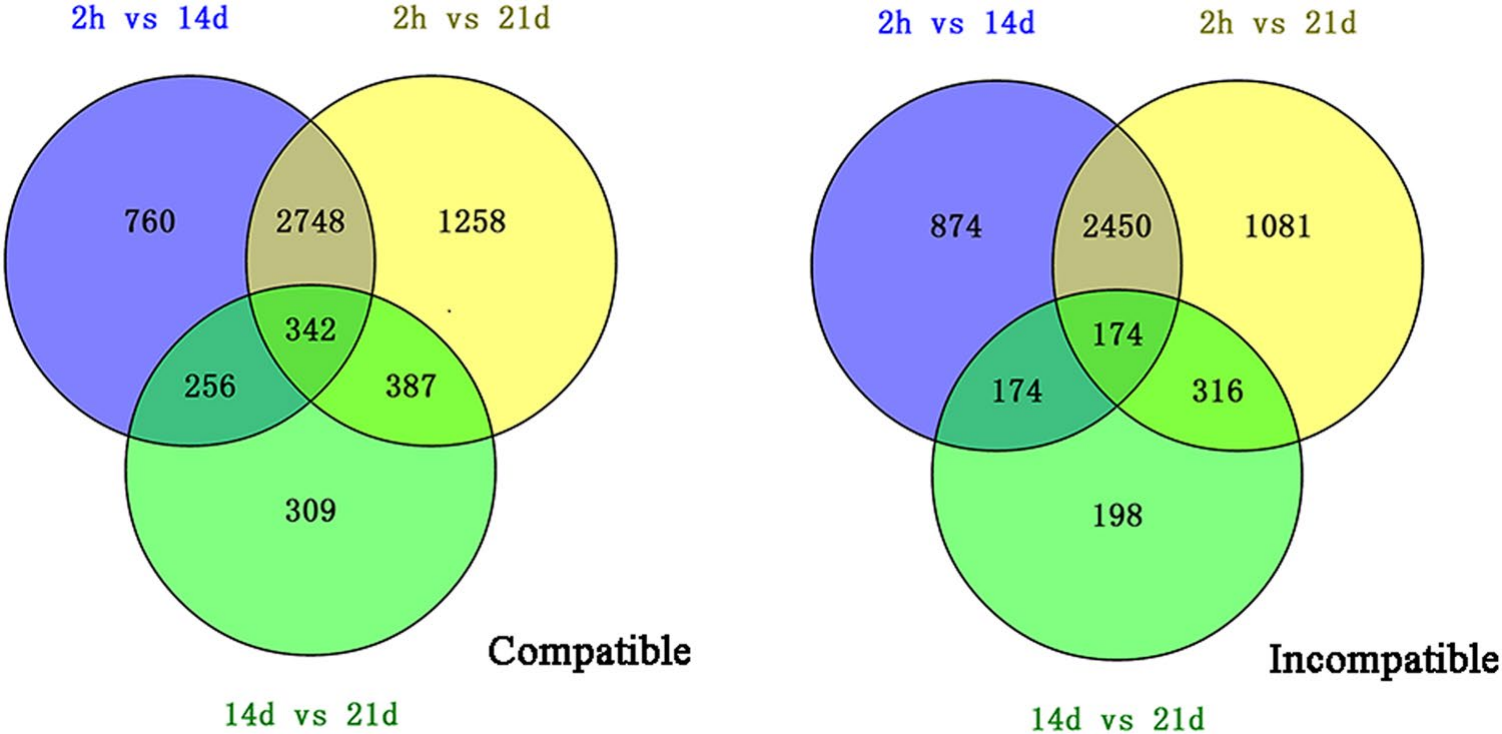
Venn diagram showing total number of the differentially expressed genes between the compatible combination and incompatible combination.

The expression patterns of transcripts between the compatible and incompatible combinations were investigated. Pair wise comparison of the graft union revealed many differentially expressed transcripts [|log_2_Ratio| ≥ 1, false discovery rate (FDR) ≤ 0.001] at 2 h, 14 d and 21 d after grafting in the graft compatibility and incompatibility (Fig. 3, Supplementary Dataset). The highest number of DEGs was noticed between 2 h and 21 d after grafting. A total of 4735 DEGs was identified between J/J 2 h vs J/J 21 d after grafting, whereas the number in J/Z graft combination was 4021. In addition, there were 342 and 174 DEGs at the three stages after grafting in J/J compatible combination and J/Z incompatible combination, respectively (Fig. 3). Further analysis of the DEGs revealed that the number of down-regulated transcripts was larger than that of the up-regulated transcripts at each stage after grafting in either compatible combination or incompatible combination (Figure S4), indicating lots of metabolic pathways were suppressed during graft union formation.

Expression patterns analysis indicated that all the DEGs in compatible and incompatible combinations could be divided into 4 groups: uptrend pattern, downtrend pattern, first increased then decreased pattern, and first decreased then increased pattern (Figure S5). In the group of downtrend pattern, the top 3 KEGG pathways were plant-pathogen interaction, plant hormone signal transduction and circadian rhythm in the compatible combination, whereas the top 3 KEGG pathways were cutin, suberine and wax biosynthesis, aminobenzoate degradation, and ascorbate and aldarate metabolism in the incompatible combination. In the group of uptrend pattern, phenylpropanoid biosynthesis was the top KEGG pathway in both compatible and incompatible combination.

### qRT-PCR validation of DEGs from transcriptome analysis

**Figure 4:**
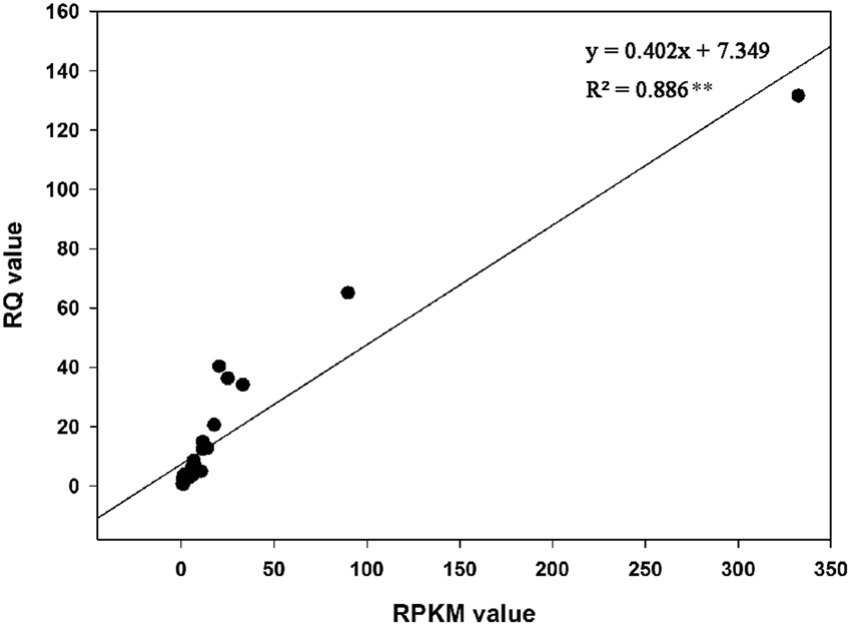
Correlation between qRT-PCR and data obtained from transcriptome analysis. The real-time PCR log_2_ values (x-axis) were plotted against graft healing stages (y-axis). **Indicates a significant difference at p ≤ 0.01.

To confirm the accuracy and reproducibility of the transcriptome analysis results, 8 genes related to lignin biosynthesis showing differential expression patterns were selected for real-time quantitative reverse transcription PCR (qRT-PCR) validation. The correlation between RNA-seq and qRT-PCR was evaluated. The results showed that there was a significantly positive correlation between the two methods (Fig. 4), thereby confirming our transcriptome analysis.

### Identification of transcription factors showing differential expression pattern between graft compatibility and incompatibility

**Figure 5:**
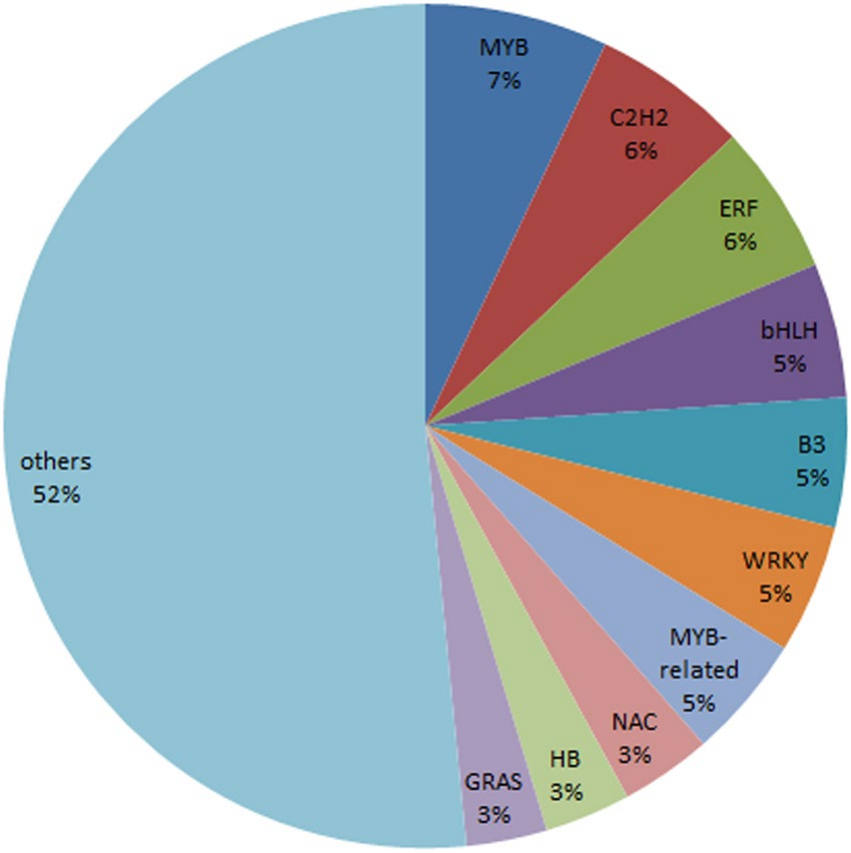
Classification of identified TFs showing differentially expressed during graft union formation.

Transcription factors (TFs) play a key role in regulating the secondary metabolism by controlling gene expression^24^. Using the iTAK pipeline V1.5 (http://bioinfo.bti.cornell.edu/tool/itak) to identify TFs and protein kinases, 30408 DEGs have a high homology index with 485 TFs of that belong to 66 known TF families (Fig. 5). Among them, 34 MYBs and 25 bHLHs was identified. Previous studies in other plants showed that these kinds of TFs regulate various metabolic pathways such as phenylpropanoid and flavonoid metabolism^25^. Moreover, there were 10 bZIPs, which may be involved in stress responses.

### Auxin signal pathway activated during graft process

**Figure 6:**
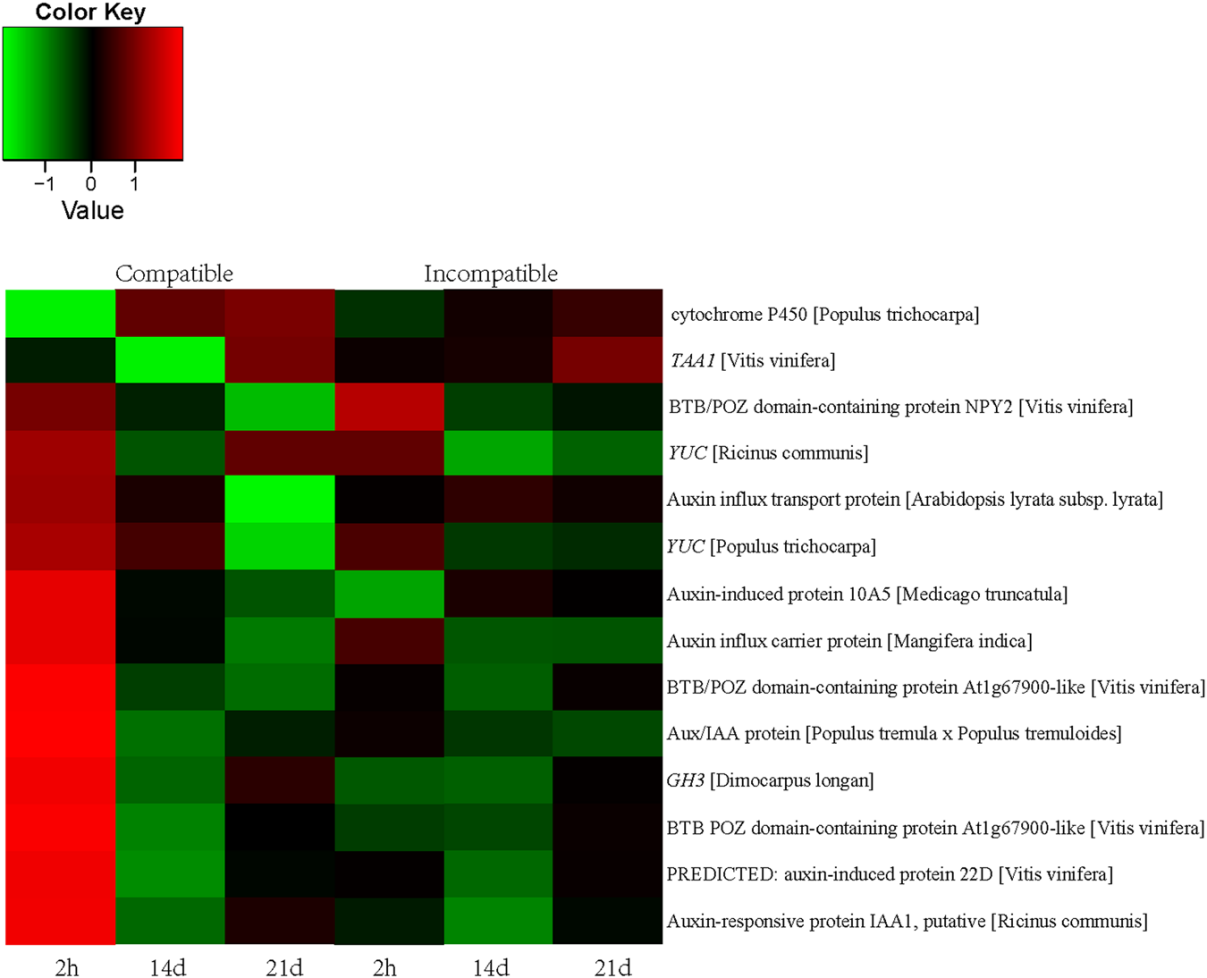
Heat map diagram of relative gene expression levels of DEGs related to auxin pathway. Red color indicates a relative increase in expression, and green color represents a relative decrease in expression.

Plant hormones used for communication between rootstock and scion were believed to be required for graft union formation^1^, among which auxin was reported to be involved in vascular formation^3^. In this study, 9 DEGs annotated as TAA, YUC, Aux/IAA, auxin-responsive protein IAA and auxin-induced protein involved in IAA signal transduction or IAA biosynthesis pathways were differentially expressed, and all were up-regulated in compatible combination initially after grafting. The expression abundance decreased significantly during the late graft stages in compatible combination, however, the expression level in incompatible combination showed low and stable trends (Fig. 6). qRT-PCR results confirmed that the expression level of these genes were higher in compatible combination than that in the incompatible combination, especially at the early stage of graft formation (Figure S6), indicating IAA might play an important role in the healing process of grafting.

**Figure 7:**
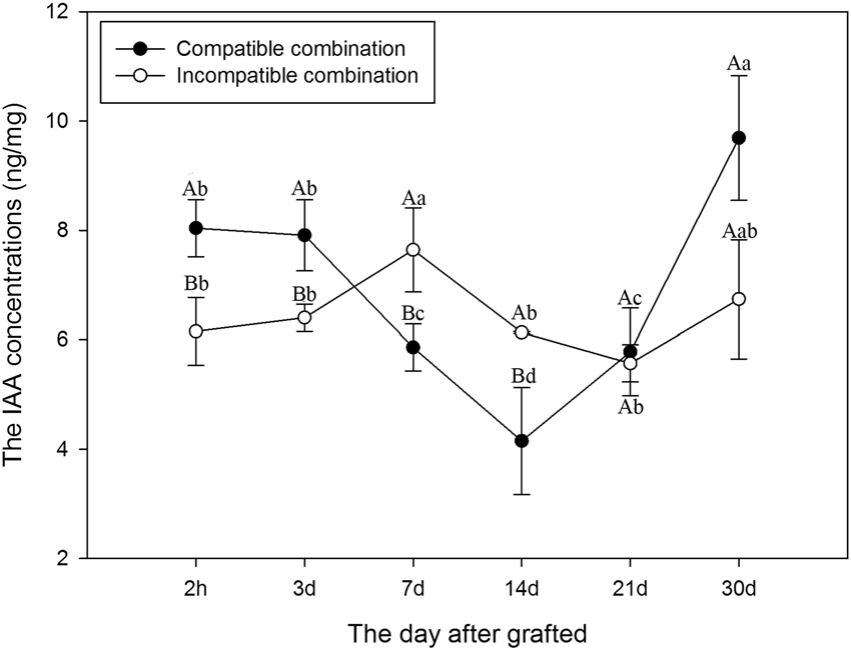
Comparison of the IAA concentrations in graft union between the compatible and incompatible combination. Data were mean ± SE of three biological replicates. Different lowercase letters indicate significant difference at the 0.05 level among 6 sampling sites, different capital letters indicate significant difference at the 0.05 level between compatible combination and incompatible combination at the same sampling site, respectively.

To confirm the results from transcriptome analysis and qRT-PCR, the IAA concentrations in graft union were compared between the compatible and the incompatible combination (Fig. 7). The IAA concentration in compatible combination was significantly higher than that in incompatible combination at 2 h after grafting, then gradually decreased, and increased to the highest level at 30 d after grafting. However, there was no significant change in incompatible combination, except a slight increase at 7 d after grafting (Fig. 7). This result confirmed the gene expression results and indicated that the IAA might promote the graft compatibility.

### Lignin biosynthesis in graft healing process

**Figure 8:**
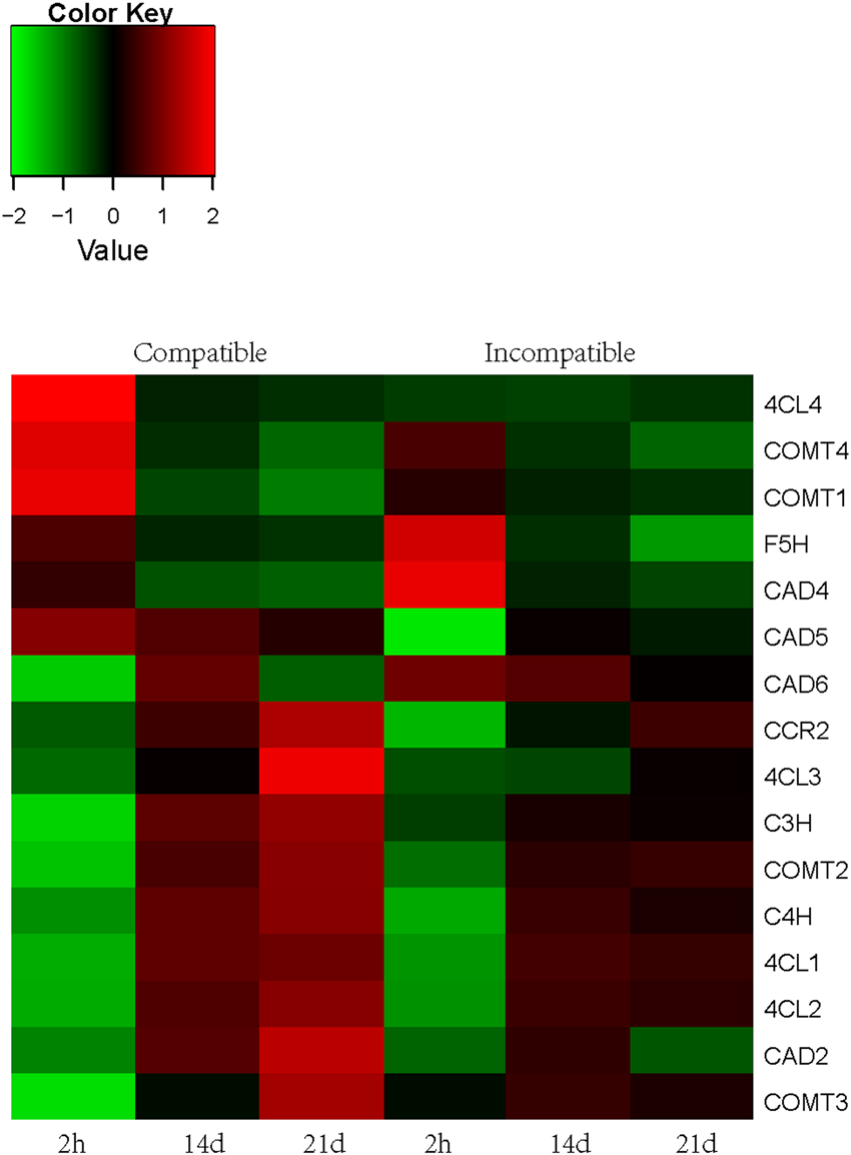
Heat map diagram of DEGs related to lignin biosynthesis in compatible and incompatible combination. Red color indicates a relative increase in expression, and green color represents a relative decrease in expression.

Lignin is the second most abundant biopolymer in nature contributing to up to 15% to 35% of the dry weight of wood^26^. In wood, lignin is polymerized from three mono lignols: the p-coumaryl, coniferyl, and sinapyl alcohols. These monolignols, when incorporated into the lignin polymer, are called the guaiacyl (G), syringyl (S), and p-hydroxyphenyl (H) units. Although researchers have studied lignin for more than a century, many aspects of its biosynthesis remain a matter of debate. 187 DEGs (Supplementary Dataset) involved in phenylpropanoid biosynthesis pathway were found. Overall 27 DEGs were identified, which were related to eight of the enzymes in the general phenylpropanoid pathway: 3 *PALs*, 2 *C4Hs*, 1 *C3H*, 4 *4CLs*, 4 *CCRs*, 4 COMTs, 1 F5H and 8 CADs. The DEGs showed increased trend during graft healing process, and the expression level in compatible combination was higher than that in incompatible combination (Fig. 8). The transcriptome analysis was also confirmed by qRT-PCR (Figure S7). Furthermore, 13 putative R2R3 MYB TFs that might regulate transcription through cis-acting AC elements of genes in the phenylpropanoid and monolignol-specific pathways^27, 28^ was also differentially expressed (data not shown).

### Signal transduction in graft healing process

To investigate the signal transduction during graft healing process in litchi, the enriched signal transduction pathways and the KEGG pathways related to signal transduction were analyzed (Table 2). 12 DEGs involved in wound response were observed. The highest transcript abundance of these DEGs occurred at 2 h after grafting, and decreased in both the compatible and incompatible combination. Moreover, these DEGs were more abundant in compatible library than that in incompatible one at 2 h after grafting (Table 2), suggesting that the wound response related genes might be activated at the early graft healing process and their expression levels were higher in compatible combination.

**Table 2:** List of DEGs related to signal transduction during graft healing process.

Gene ID	Compatibility (RPKM)			Incompatibility (RPKM)			Annotation
	2 h	14 d	21 d	2 h	14 d	21 d	
Response to wound							
Litchi_GLEAN_10052959	3019.83	462.24	313.63	972.76	466.99	256.59	senescence-associated protein [Vitis quinquangularis]
Litchi_GLEAN_10048341	1159.89	147.86	244.78	68.08	131.61	185.93	mitogen-activated protein kinase [Citrus sinensis]
Litchi_GLEAN_10016027	725.94	550.77	559.42	498.68	635.45	454.27	CBL-interacting serine/threonine-protein kinase, putative [Ricinus communis]
Litchi_GLEAN_10065008	513.59	254.83	248.32	445.90	283.99	283.29	DNA binding protein, putative [Ricinus communis]
Litchi_GLEAN_10033568	393.4	50.85	80.68	68.15	48.87	81.29	gchitinase, putative [Ricinus communis]
Litchi_GLEAN_10003315	361.29	48.00	38.91	348.34	83.39	36.71	iaa-amino acid hydrolase 11 [Populus trichocarpa]
Litchi_GLEAN_10021109	283.42	54.08	62.50	18.21	47.67	32.64	CBL-interacting serine/threonine-protein kinase, putative [Ricinus communis]
Litchi_GLEAN_10027663	262.14	169.84	196.50	182.59	120.86	175.14	probable CCR4-associated factor 1 homolog 9-like [Vitis vinifera]
Litchi_GLEAN_10027663	262.14	169.84	196.50	182.59	120.86	175.14	CBL-interacting serine/threonine-protein kinase, putative [Ricinus communis]
Litchi_GLEAN_10063684	213.37	10.53	13.39	43.39	9.41	7.59	1,2-diacylglycerol 3-beta-galactosyltransferase, putative [Ricinus communis]
Litchi_GLEAN_10008363	153.28	13.98	27.91	6.14	23.85	28.35	12-oxophytodienoate reductase 3-like [Glycine max]
Litchi_GLEAN_10019124	141.9	116.46	119.18	62.17	98.74	94.07	receptor protein kinase, putative [Ricinus communis]
CaM							
Litchi_GLEAN_10049763	120.57	16.92	30.48	7.61	15.50	20.02	calcium-dependent protein kinase, putative [Ricinus communis]
Litchi_GLEAN_10061686	111.23	61.68	70.65	69.94	59.01	63.20	calcium-dependent protein kinase, putative [Ricinus communis]
Litchi_GLEAN_10030665	97.29	25.69	21.91	25.59	26.71	24.87	calmodulin-binding protein [Populus euphratica]
Litchi_GLEAN_10034079	92.61	82.50	82.48	76.04	85.04	75.51	calcium-dependent protein kinase [Gossypium hirsutum]
Litchi_GLEAN_10015725	58.94	15.07	20.14	21.46	18.12	20.21	calcium-dependent protein kinase [Dimocarpus longan]
Litchi_GLEAN_10007743	38.48	30.57	58.89	25.38	28.24	43.27	calmodulin-binding transcription activator 2-like [Cucumis sativus]
Litchi_GLEAN_10032706	30.35	14.01	17.58	20.41	17.66	19.79	calcium-dependent protein kinase, putative [Ricinus communis]
Litchi_GLEAN_10063533	21.85	14.20	14.21	16.62	15.04	16.48	calcium dependent protein kinase 14 [Populus trichocarpa]
Litchi_GLEAN_10019427	10.82	8.95	18.44	5.89	9.41	13.35	calcium-transporting ATPase 12, plasma membrane-type-like [Vitis vinifera]
MAPK							
Litchi_GLEAN_10048341	1159.89	147.86	244.78	68.08	131.61	185.93	mitogen-activated protein kinase [Citrus sinensis]
Litchi_GLEAN_10048356	518.64	55.97	69.86	41.64	48.23	54.92	big map kinase/bmk, putative [Ricinus communis]
Litchi_GLEAN_10039455	134.69	61.37	58.65	65.38	59.81	72.16	big map kinase/bmk, putative [Ricinus communis]
Litchi_GLEAN_10030350	93.71	43.64	47.47	41.79	40.98	46.98	Mitogen-activated protein kinase kinase kinase, putative [Ricinus communis]
Litchi_GLEAN_10045161	59.61	4.92	8.19	58.27	5.75	10.45	kinase, putative [Ricinus communis]
Litchi_GLEAN_10055953	40.63	35.87	45.82	32.16	36.68	42.48	mitogen-activated protein kinase 9-like [Vitis vinifera]
Litchi_GLEAN_10058235	39.07	13.87	15.43	17.16	11.06	10.34	mitogen-activated protein kinase MAPK [Prunus armeniaca]
Litchi_GLEAN_10031819	24.43	6.79	7.48	5.01	6.20	5.30	kinase, putative [Ricinus communis]
Litchi_GLEAN_10028531	15.73	12.23	14.42	13.16	12.88	13.90	map3k delta-1 protein kinase, putative [Ricinus communis]
Litchi_GLEAN_10058386	12.61	2.41	5.29	5.97	3.11	4.12	kinase, putative [Ricinus communis]
Litchi_GLEAN_10047600	12.34	1.10	0.94	2.79	1.87	1.27	Mitogen-activated protein kinase kinase kinase, putative [Ricinus communis]

In addition to wound response, DEGs involved in other signal transduction pathways were also identified (Table 2). CaM and MAPK pathways were highly enriched in KEGG pathways. DEGs annotated as calcium-dependent protein kinase, calmodulin-binding protein and mitogen-activated protein kinase increased and became more abundant in compatible combination at the 2 h after grafting than that in incompatible one.

## Discussion

Graft healing procedure is a critical and complicated plant developmental process influenced by numerous endogenous and environmental factors. In graft union development, substantial changes occur, such as cell differentiation and organ regeneration, and even genetic material can be exchanged between the scion and stock at the graft site^29^. However, the molecular mechanisms of graft union formation remain unclear. In the present study, transcriptome changes between compatible and incompatible graft combination of litchi by digital gene expression were investigated, providing the first view of the complex network regulating graft compatibility in litchi.

### Digital gene expression profile during graft healing process

Next generation sequencing (NGS) is proved to be of great value for functional genomic study of non-model species^30^. In this study, RNA-Seq technology was used to profile the litchi graft union transcriptome on the Illumina HiSeq^TM^ 2000 platform, and approximately 72 million reads were obtained in total. A total of 6060 DEGs were identified among the three stages in compatible combination and 5267 DEGs were identified in incompatible combination, many of which were functionally annotated. The amount of information obtained from RNA-Seq analysis was, not surprisingly, much larger than that generated using a cDNA -AFLP approach, where only 49 genes were identified from hickory during graft healing process^9^.

Functional categorization of these DEGs revealed that the grafting triggered the expression of a large number of transcripts involved in wound response, stress response, plant hormone signal transduction, metabolic process and phenylpropanoid biosynthesis. Plant grafting is presumably perceived as a considerable stress^11^. As a stress situation for plants, there was an oxidative burst at the graft interface. Oxidative stress has been implicated in the graft incompatibility response of pear/quince heterografts^31^. In our previous study, the activities of antioxidant enzymes (POD, SOD and CAT) were higher in compatible combination than that in incompatible combination. These antioxidant enzymes might involve in coping with oxidative stress and achieving successful grafting^13^. Our study here indicated that grafting upregulated wound response and antioxidant activity at the transcriptional level. DEGs involved in oxidative stress in the heterograft and autograft of grapevine was also reported by Cookson *et al*.^11^. Moreover, DEGs related to CaM or MAPK which are signal molecules were up-regulated at the early stage during graft healing process. The roles of these signal molecules in graft union formation are deserved to be further studied.

A common theme to plant wound responses is the involvement of plant hormones, which play important roles in plant growth and development^3^. Studies indicated that auxin is important in vascular development^32, 33^. Yin *et al*.^10^ found that cell division commenced after auxin accumulation using a micrografting method in *Arabidopsis*. Our GC-MS results indicated that the IAA concentration in graft union was enhanced immediately after grafting, the same results were also observed at the early stage of hickory grafting^9^. Gene expression changes during graft healing process in hickory revealed that the genes related to IAA transport were differentially expressed^9^. The transport and release of IAA during graft union formation could induce the expression of auxin-response genes, thereby stimulating cell-elongation growth^34^. Our RNA–seq data showed that IAA related genes were elevated at 2 h after grafting. IAA signal exchange after wounding is indispensable for subsequent events during graft-union healing^10^. It is conceivable that similar mechanisms activated by IAA after wounding may play a part in the early responses of both graft parts in litchi. The auxin accumulation and auxin-response genes expression remind us of the importance of auxin during the signal-exchange process between scions and rootstocks. Recently, Melnyk *et al*.^3^ proposed an inter-tissue communication process that occurred at the graft junction and promoted vascular connection by tissue-specific auxin responses involving ALF4. However, the roles of auxin in the vascular connection during litchi grafting need further analyzed.

Cellulose and lignin are two important biopolymers in the vascular bundle, and successful grafting in plants requires the development of a functional vascular system between scion and rootstock. According to the currently accepted lignin metabolic pathway, almost all genes required to encode the related enzymes were found in our transcriptome data set. Many of the genes appear to be from multigene families, which is consistent with related reports of *Arabidopsis* ^35^ and poplar^36^. In the present study, the lignin biosynthesis related genes were upregulated at 21 d after grafting in the compatible combination. In previous study, cell-wall precursor and lignin biosynthesis were also enriched in the grapevine heterograft compared to the autograft control due to differences in plant wounding and defense responses^11^. The upregulation of *PAL* gene expression has also been reported during the graft union formation^37, 38^. The expression of *ParPAL1* from *Prunus* were differentially expressed between compatible and incompatible unions throughout graft development, and *ParPAL1* transcripts were 3- fold more abundant in incompatible union than those of compatible one^38^. The expression of *PAL* gene in litchi was also differentially expressed between compatible and incompatible unions (data no shown).

In this study, 259 TFs were differentially expressed during graft healing process and, of particular note, 12 *WRKY* and 8 *NAC* were significantly differentially expressed. The NAC family is one of the largest plant-specific TF families with diverse roles in plant development and stress regulation^39^. The WRKY TFs participate in various plant defense responses and regulate plant growth and development^40^. As we known, grafting for plant is a kind of stress. We propose that these TFs are involved in graft healing process.

### Transcriptome changes between compatible and incompatible graft combinations

Graft incompatibility is defined as failure of the graft combination to form a strong and lasting functional graft union^2^. Yet, despite the numerous studies on grafting dealing with compatible/incompatible union development in vegetables^41^ and fruits^42–45^, the physiological and molecular mechanisms involved in graft incompatibility are still vague^2^. In the present study, the transcriptome changes between compatible and incompatible graft combinations in litchi were investigated. Based on the functional categorization of DEGs, grafting induced genes involved in wound response, auxin signal transduction, and metabolic process, which may play key roles in vascular differentiation. This hypothesis is supported by the recent study of Melnyk *et al*.^3^ who demonstrated the involvement of auxin in graft union formation and vascular reconnection in *Arabidopsis*.

The number of DEGs related to stress response in compatible combination was higher than that in incompatible combination. The largest difference of genes expression profiling between compatible and incompatible combinations occurred at the early stage of graft union formation (2 h after grafting). It is worth noting that the phenylpropanoid biosynthesis pathway may play important roles in graft compatibility/incompatibility. Moreover, the gene involved in metabolic pathways was higher enriched in compatible combination than that in incompatible combination. These DEGs or pathways would provide some clues in understanding the mechanisms involved in the graft incompatibility.

## Conclusions

**Figure 9:**
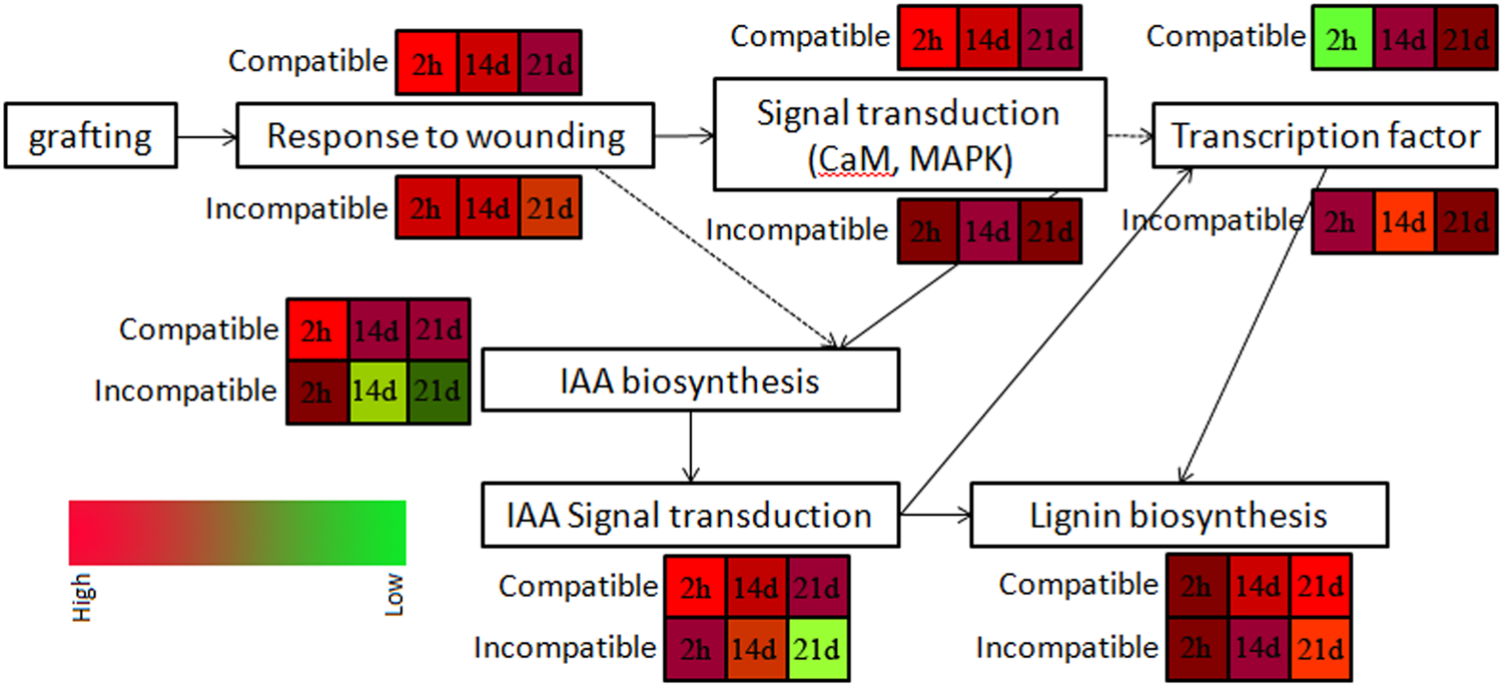
A hypothetical model of graft-union development in compatible and incompatible grafting of litchi. Different colors represent the expression levels of key genes in each pathway of the litchi compatible graft J/J and incompatible graft J/Z at 2 h, 14 d, and 21 d after grafting.

Our transcriptome analysis of the graft interfaces of compatible homograft and incompatible heterograft of litchi 2 h, 14 d and 21 d after grafting revealed interesting genes that might be involved in graft healing process. Moreover, a comparative analysis of the DEGs between compatible homograft and incompatible heterograft during graft union formation provided some clues revealing the mechanisms of graft incompatibility. A hypothetical model of graft-union development in compatible and incompatible grafting of litchi was proposed (Fig. 9). Genes related to wound response were immediately upregulated after grafting, then auxin and signal transduction pathways (such as CaM and MAPK) were induced. Finally, the TFs regulated the lignin biosynthesis and promoted vascular reconnection between the scion and rootstock. DEGs related to stress response, auxin and signal transduction at early stage of grafting might determine graft compatibility or incompatibility.

## Materials and Methods

### Plant materials and grafting

*Litchi chinensis* cv. ‘Jingganghongnuo’ and ‘zhuangyuanhong’ used in the present study were planted in the experimental orchard of South China Agricultural University, Guangzhou, China. ‘Jingganghongnuo’ grafted onto ‘zhuangyuanhong’ (heterograft) as incompatible combination^13^, and ‘Jingganghongnuo’ grafted onto itself (homograft) as compatible combination. Tender branches were collected from the mature trees of litchi for splice grafting. The rootstocks were 10 years old which were considered to be in the adult phase. Each combination was grafted 100 times. Trees used in the experiment were not pruned or chemically treated during the experimental period. The grafting survival rate defined as the percent of plants survived the grafting was calculated 6 months after grafting. All samples were collected from the graft interfaces of compatible homograft and incompatible heterograft on 2 h, 14 d and 21 d after grafting. Samples were stored immediately in liquid nitrogen and then stored at −80 °C until use.

### Paraffin section microscopy

The samples were collected on 2 h, 7 d, 14 d, 21 d, 32 d, 45 d after grafting and fixed in 2.5% glutaraldehyde in 0.03 M phosphate buffer above 24 h^46^. Then the samples were softened in glycerol: alcohol mixture liquid (1:1) about 1 week and dehydrated in an ethanol series (15%, 30%, 50%, 70% and 95%), each dehydrated steps for 90 min. After infiltration in Safranin overnight and decoloration by using dimethylbenzene, the specimens were embedded in paraffin. Samples were sectioned on a sliding microtome at a thickness of 10 μm. Finally, the paraffin sections were observed using the Zeiss photomicroscope II (Carl Zeiss Jena, Germany).

### RNA extraction, library construction and RNA-Sequencing

Total RNA was extracted using the Quick RNA Isolation Kit (Huayueyang, China) according to the manufacturer’s instruction and treated with DNase I (TaKaRa, Japan) to remove genomic DNA contamination. Then the mRNA is enriched by using the oligo (dT) magnetic beads (for eukaryotes). Each library was pooled by mixing equal quantities of RNA from three biological replications for each stage. The steps of mRNA enrichment, mRNA fragmentation, second-strand cDNA synthesis, size selection, PCR amplification and subsequent sequencing using an Illumina HiSeq™ 2000 (San Diego, CA, USA) were performed at the Beijing Genome Institute (BGI, Shenzhen, China).

### Bioinformatics analysis

Raw reads were filtered to remove low quality tags (tags with unknown sequences ‘N’), empty tags (sequence with only the adaptors but no tags), and tags with only one copy number (probable sequencing error). Clean reads were mapped to the reference sequences (litchi non-redundancy genome) using SOAP aligner/SOAP2^47^. No more than 2 mismatches were allowed in the alignment. All tags that mapped to reference sequences of multiple genes were filtered out and the remaining tags were designated as unambiguous tags for gene expression analysis. The number of unambiguous tags of each gene was calculated and then normalized to RPKM (Reads per kb per Million reads).

To compare the differently expressed genes, the frequency of each tag in different digital gene expression libraries was statistically analyzed using the method of Audic & Claverie^23^. The false discovery rate (FDR) was used to determine the threshold *P*-value in multiple tests. We used FDR ≤ 0.001 and the absolute value of log_2_Ratio ≥1 as the threshold to judge the significance of gene expression difference.

Functional annotation of the differentially expressed genes was predicted based on the highest similarity in the following databases: Nr (NCBI non-redundant protein sequences, http://www.ncbi.nlm.nih.gov), Nt (NCBI non-redundant nucleotide sequences, http://www.ncbi.nlm.nih.gov), COG (Clusters of Orthologous Groups of proteins, http://www.ncbi.nlm.nih.gov/COG), KO (KEGG Orthology database, http://www.genome.jp/kegg) and GO (Gene Ontology). GO functional annotation was performed by Blast2GO (v2.5.0) software^48^.

### Quantitative real-time PCR analysis

Total RNA was isolated as described above and reverse transcribed with oligo (dT)_18_ primers using M-MLV reverse transcriptase (Invitrogen, USA) according to the manufacturer’s recommendations. Transcript levels were analyzed using quantitative RT-PCR with the DyNAmo Flash SYBR Green qPCR kit (Thermo, USA) and the CFX96 qPCR System (Bio-Rad, USA) according to the manufacturer’s instructions. Gene-specific primers were designed using the Primer 5.0 program (PREMIER Biosoft International, Canada) (Table S1). All reactions were performed in triplicate with three biological replicates. All reactions were normalized using the Ct values corresponding to *Lcactin* gene (HQ615689). Unigene expression levels were calculated using the 2^ΔΔCt^ method^49^.

### Quantitative analysis of indole-3-acetic acid

GC-MS was used to determine the concentrations of indole-3-acetic acid (IAA) of the graft interfaces of compatible/incompatible combinations. Abundant cold methanol (80%, v/v) was used to extract IAA, and internals standards, 2H-IAA 100 ng were simultaneously introduced into the samples. Crude extract was condensed by vacuum evaporating and hormones were re-extracted by ethyl acctate at pH 2.5–3.0. Then condensed IAA extracts were purified by Sep-Pak C18 columns. Thereafter, purified hormones were methylated using trimethylsilyldizomethane, then analyzed by GC-MS after an additional TMS reaction.

### Statistical analysis

Statistical analyses were performed with SPSS software (SPSS, Chicago, IL). One-way analysis of variance (ANOVA) was used to evaluate the Unigene expression levels on the different graft combinations. Two-way repeated measures anova was used to compare IAA concentrations in compatible and incompatible combinations at different time points. Heatmap diagram were analyzed with pheatmap methods using R software. Significant correlations between qRT-PCR and data obtained from transcriptome analysis were analyzed with SPSS software using Pearson’s correlation as the statistical metric. Significant correlations were considered only when an adjusted *P* value was lower than 0.05.

### Availability of supporting data

The clean data for the digital gene expression analysis were also deposited in the NCBI Sequence Read Archive under accession numbers SRR5313174 (JGHN_ZYH21d), SRR5313175 (JGHN_ZYH14d), SRR5313176 (JGHN_ZYH2h), SRR5313177 (JGHN_JGHN21d), SRR5313178 (JGHN_JGHN14d), and SRR5313179 (JGHN_JGHN2h).

## Electronic supplementary material

Supplementary PDF File

Supplementary XLS File
