# Supplementary material for: Transcriptome changes between compatible and incompatible graft combination of Litchi chinensis by digital gene expression profile
Source: Sci Rep. 2017 Jun 21;7:3954. doi: 10.1038/s41598-017-04328-x (PMC5479835; doi:10.1038/s41598-017-04328-x)
Supplement: Supplementary file 1 — Supplementary PDF File [file 41598_2017_4328_MOESM1_ESM.pdf]

Transcriptome changes between compatible and incompatible graft combination of *Litchi chinensis* by digital gene expression profile

Zhe Chen[a, b, c], Jietang Zhao[a, b], Fuchu Hu[a, b, c], Yonghua Qin[a, b], Xianghe Wang[c], Guibing Hu[a, b] *

Supplementary materials

Table S1 Gene-specific primers for qRT-PCR analysis.

Figure S1 The survival rate and growth vigor between J/J and J/Z graft combination.

Figure S2 GO assignment of DEGs in graft compatibility (A) and incompatibility (B).

Figure S3 KEGG pathway enrichment analysis in graft compatibility (A) and incompatibility (B).

Figure S4 The numbers of up- or down-regulated DEGs between J/J and J/Z graft combination.

Figure S5 Expression pattern analysis of differentially expressed genes.

Figure S6 qRT–PCR analysis of selected DGEs related to auxin pathway. *Lcactin* was used as reference gene to normalize gene expression levels under identical conditions. The vertical bars represent the standard error of triplicate experiments.

Figure S7 qRT–PCR analysis of DGEs related to lignin biosynthesis in compatible and incompatible combination. *Lcactin* was used as reference gene to normalize gene expression levels under identical conditions. The vertical bars represent the standard error of triplicate experiments.

Table S1 Gene-specific primers for qRT-PCR analysis

Gene names	Primers
4CL1	5′-AACCAACATAGAAGGGAACAG-3′
	5′-TTGACGAGTTTAGTGGCTGA-3′
4CL2	5′-CCAGTGTTGGAAGCCTATG-3′
	5′-TTGACTCCTGCCTCTTGTG-3′
4CL3	5′-GGTAAATCTCTGCCAAGGAA-3′
	5′-TCAATGTAGCCAATGTCACC-3′
C3H1	5′-AACAGTAGAGTGGGCAATGG-3′
	5′-GCAACCTCAGACCTTCCTT-3′
C4H1	5′-GTGGGAGGAAGAGATGGAG-3′
	5′-CCTGAATGAACAAAGGGTCT-3′
CCR2	5′-GTGGTTGCTGCTCACATTT-3′
	5′-TTCTTGCTTGCTACACTTGG-3′
COMT1	5′-ATGATTGGAGCGACGAAC-3′
	5′-AGCCGCAACTGGAAGTATT-3′
COMT2	5′-TGAACCAGTGTTGCCTAATG-3′
	5′-CTTCTTCGGTCCTGTGCTT-3′
COMT3	5′-ATTGAACTTGGTGTGCTTGA-3′
	5′-TAGCGAGAAGGCGAAGAAT-3′
COMT4	5′-CACCCTTGATGCTTGACC-3′
	5′-GGGACCTAATGACACACCAT-3′
F5H	5′-ATCCACCAATCCCTCTTCTC-3′
	5′-AAATCAGGCACACCTTCTTG-3′
CAD2	5′-TGGAGGCTACTCTGACACAA-3′
	5′-CAACCCAACTACACCCACAT-3′
CADF4	5′-AAATCACCCGAAGAAGAACA-3′
	5′-ATCAGAGTGGCAAACACCA-3′
CADF5	5′-TAGTGGGTCTGCCTGAGAAG-3′

	5′-CAATGTCTGCCCTTATGTTG-3′
CADF6	5′-AGAAAGAGGAAGCCCTGAGT-3′
	5′-GCACCGATAATGAGACTTGC-3′

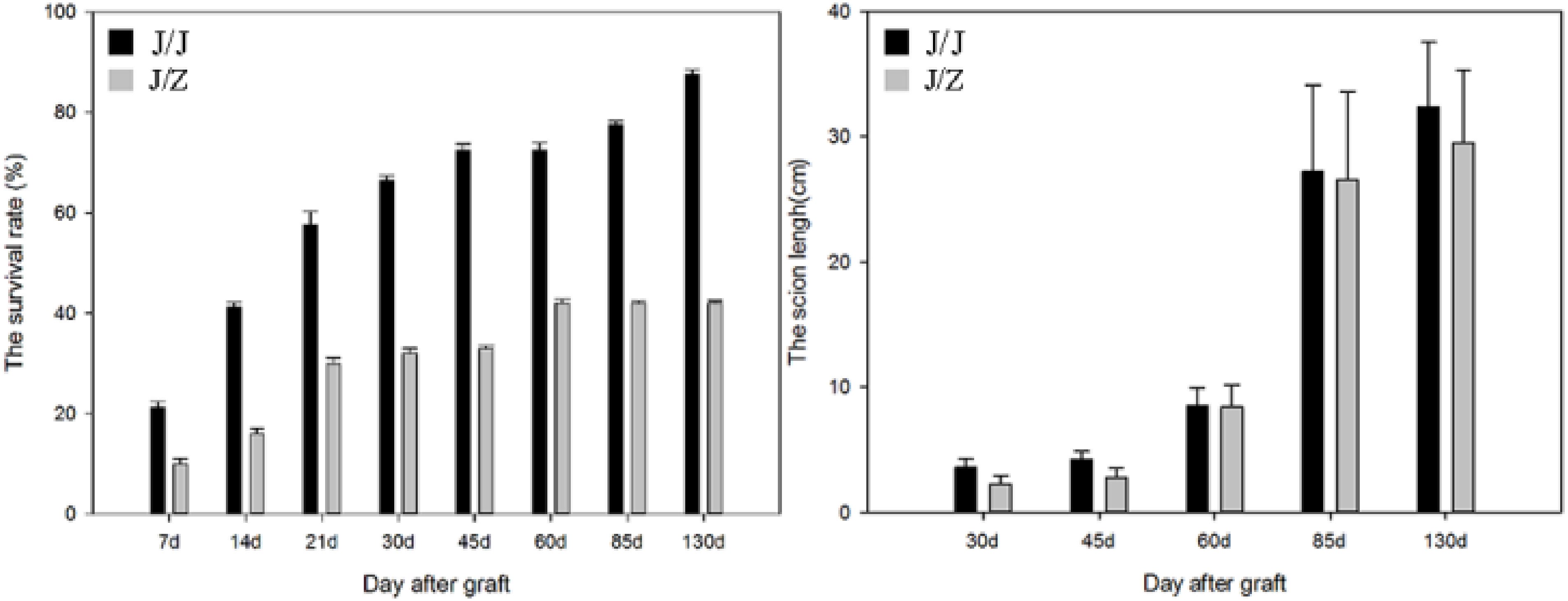

J/J
J/Z
The survival rate (%)
100
80
60
40
20
0
7d
14d
21d
30d
45d
60d
85d
130d
Day after graft
J/J
J/Z
The scion lengh(cm)
40
30
20
10
0
30d
45d
60d
85d
130d
Day after graft

Figure S1

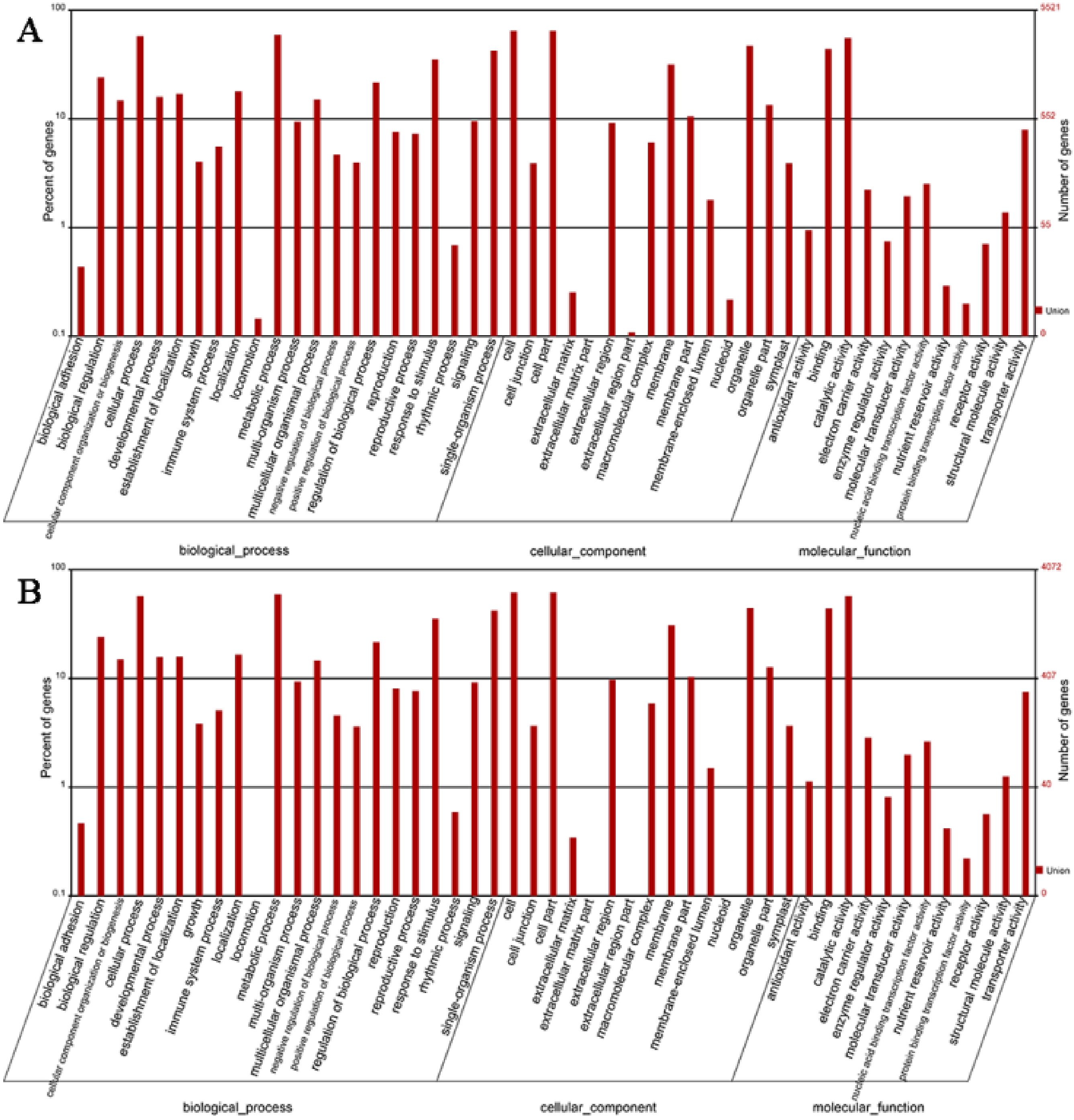
A
B
Percent of genes
Number of genes
100
10
1
0.1
5521
552
55
0
4072
407
40
Union
biological adhesion
biological regulation
cellular component organization or biogenesis
cellular process
developmental process
establishment of localization
growth
immune system process
localization
locomotion
metabolic process
multi-organism process
multicellular organismal process
negative regulation of biological process
positive regulation of biological process
regulation of biological process
reproduction
reproductive process
response to stimulus
rhythmic process
signaling
single-organism process
cell
cell junction
cell part
extracellular matrix
extracellular matrix part
extracellular region
extracellular region part
macromolecular complex
membrane
membrane part
membrane-enclosed lumen
nucleoid
organelle
organelle part
symplast
antioxidant activity
binding
catalytic activity
electron carrier activity
enzyme regulator activity
molecular transducer activity
nucleic acid binding transcription factor activity
nutrient reservoir activity
protein binding transcription factor activity
receptor activity
structural molecule activity
transporter activity
biological_process
cellular_component
molecular_function

Figure S2

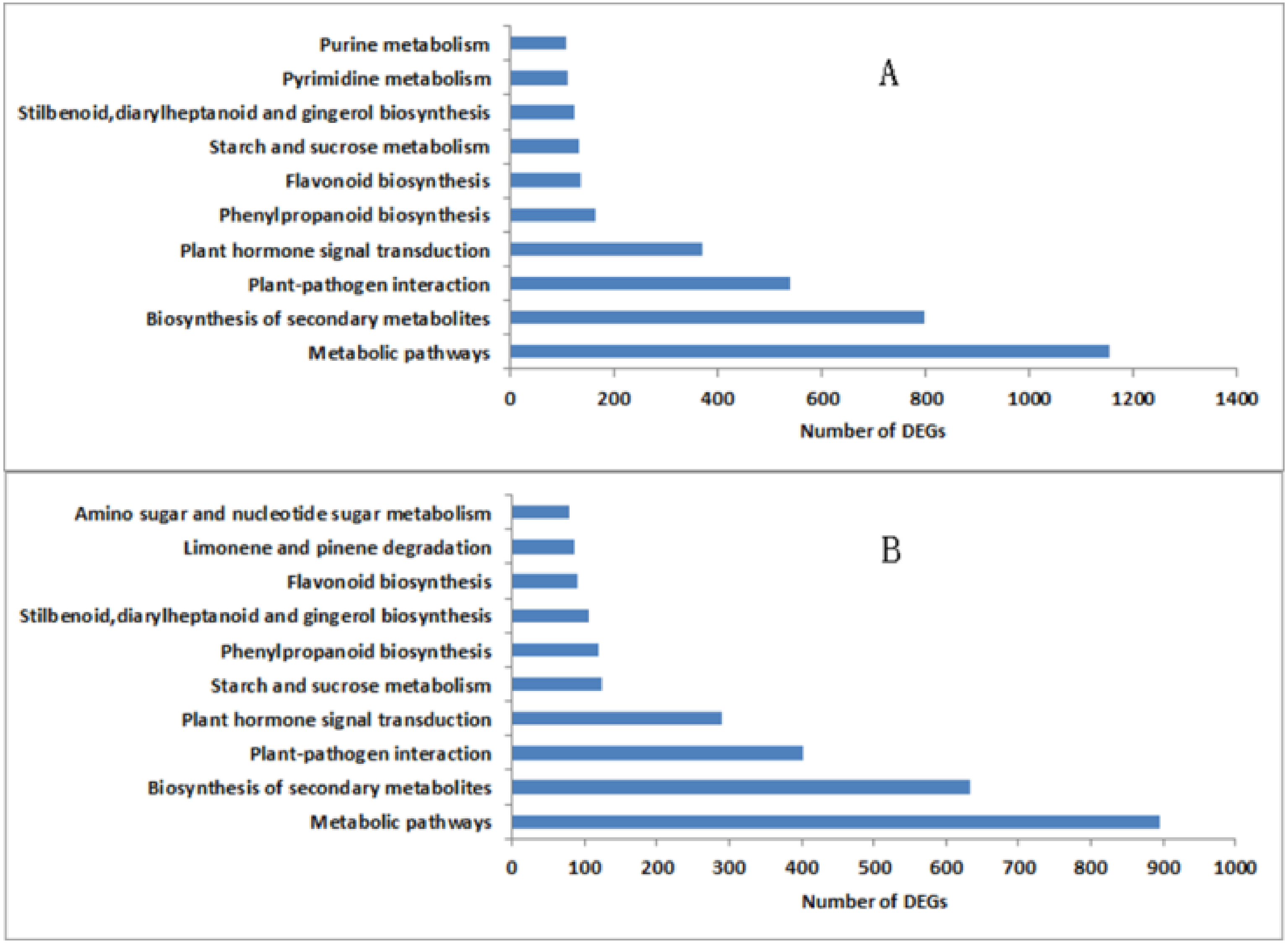
A
Purine metabolism
Pyrimidine metabolism
Stilbenoid, diarylheptanoid and gingerol biosynthesis
Starch and sucrose metabolism
Flavonoid biosynthesis
Phenylpropanoid biosynthesis
Plant hormone signal transduction
Plant-pathogen interaction
Biosynthesis of secondary metabolites
Metabolic pathways
0 200 400 600 800 1000 1200 1400
Number of DEGs
B
Amino sugar and nucleotide sugar metabolism
Limonene and pinene degradation
Flavonoid biosynthesis
Stilbenoid, diarylheptanoid and gingerol biosynthesis
Phenylpropanoid biosynthesis
Starch and sucrose metabolism
Plant hormone signal transduction
Plant-pathogen interaction
Biosynthesis of secondary metabolites
Metabolic pathways
0 100 200 300 400 500 600 700 800 900 1000
Number of DEGs

Figure S3

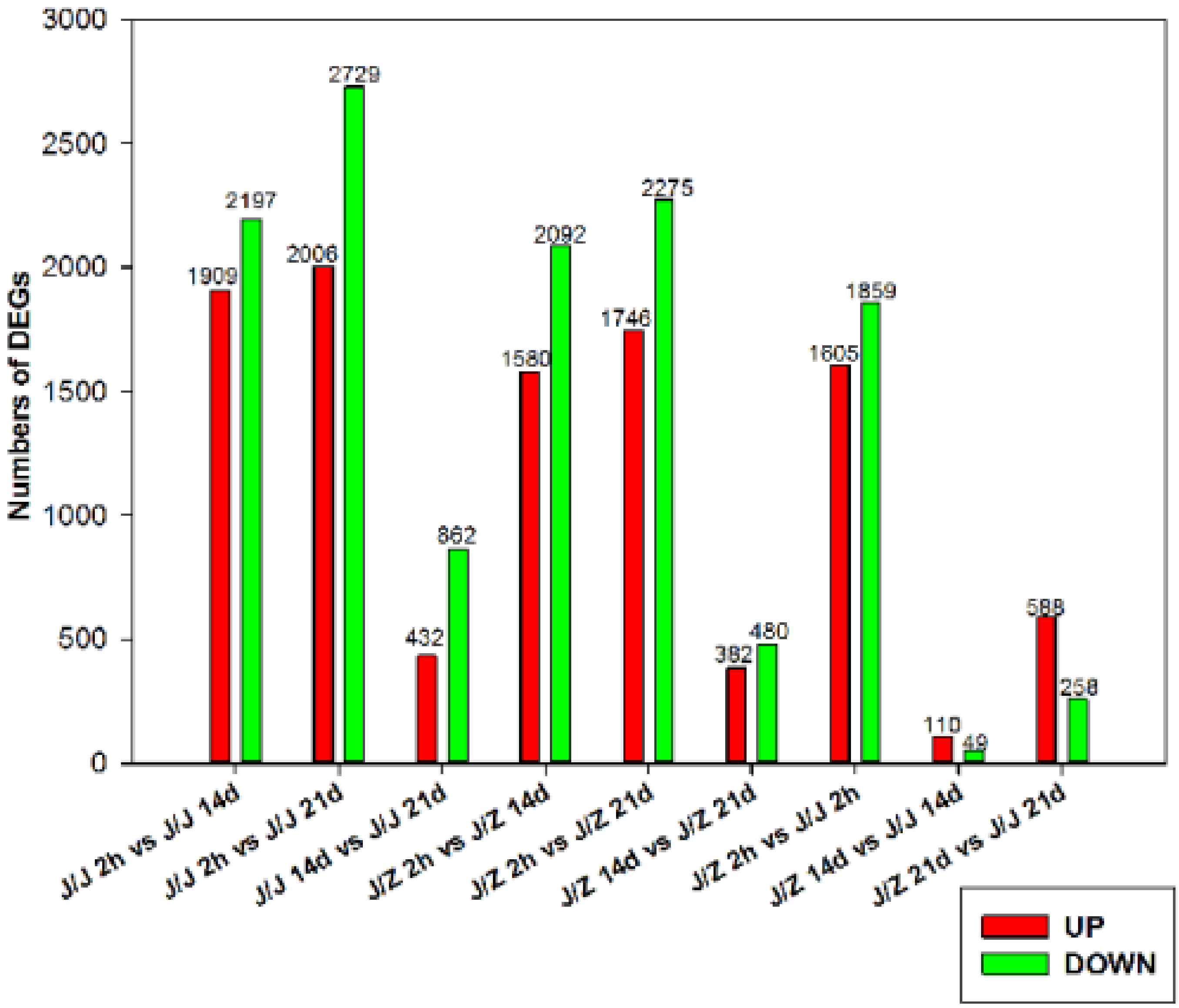

Numbers of DEGs
3000
2500
2000
1500
1000
500
0
1909
2197
2006
2729
432
862
1580
2092
1746
2275
382
480
1605
1859
110
49
588
258
J/J 2h vs J/J 14d
J/J 2h vs J/J 21d
J/J 14d vs J/J 21d
J/Z 2h vs J/Z 14d
J/Z 2h vs J/Z 21d
J/Z 14d vs J/Z 21d
J/Z 2h vs J/J 2h
J/Z 14d vs J/J 14d
J/Z 21d vs J/J 21d
UP
DOWN

Figure S4

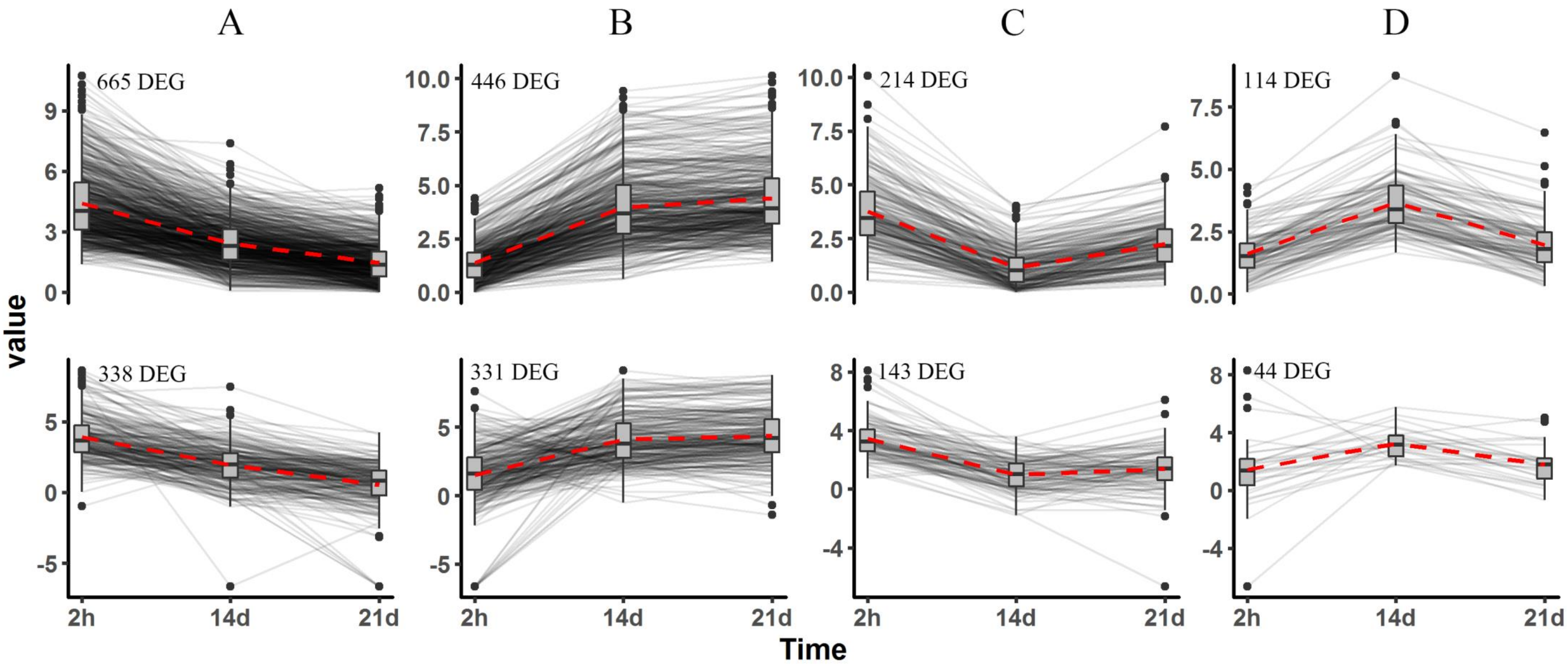

A
B
C
D
665 DEG
446 DEG
214 DEG
114 DEG
338 DEG
331 DEG
143 DEG
44 DEG
value
Time
2h
14d
21d

Figure S5

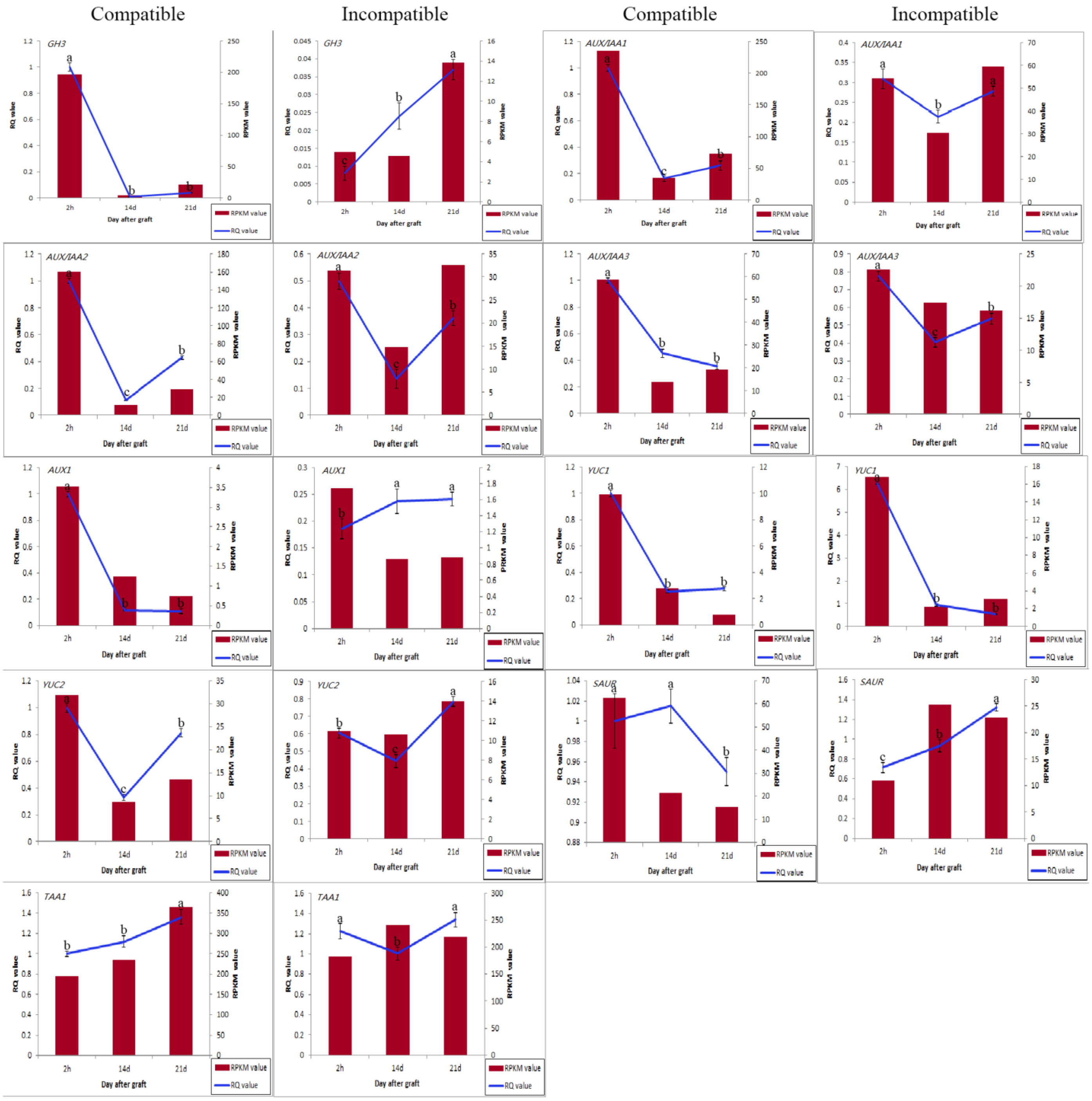
Compatible
Incompatible
Compatible
Incompatible
GH3
AUX/IAA1
AUX/IAA2
AUX/IAA3
AUX1
YUC1
YUC2
SAUR
TAA1
RQ value
RPKM value
Day after graft
2h
14d
21d

Figure S6

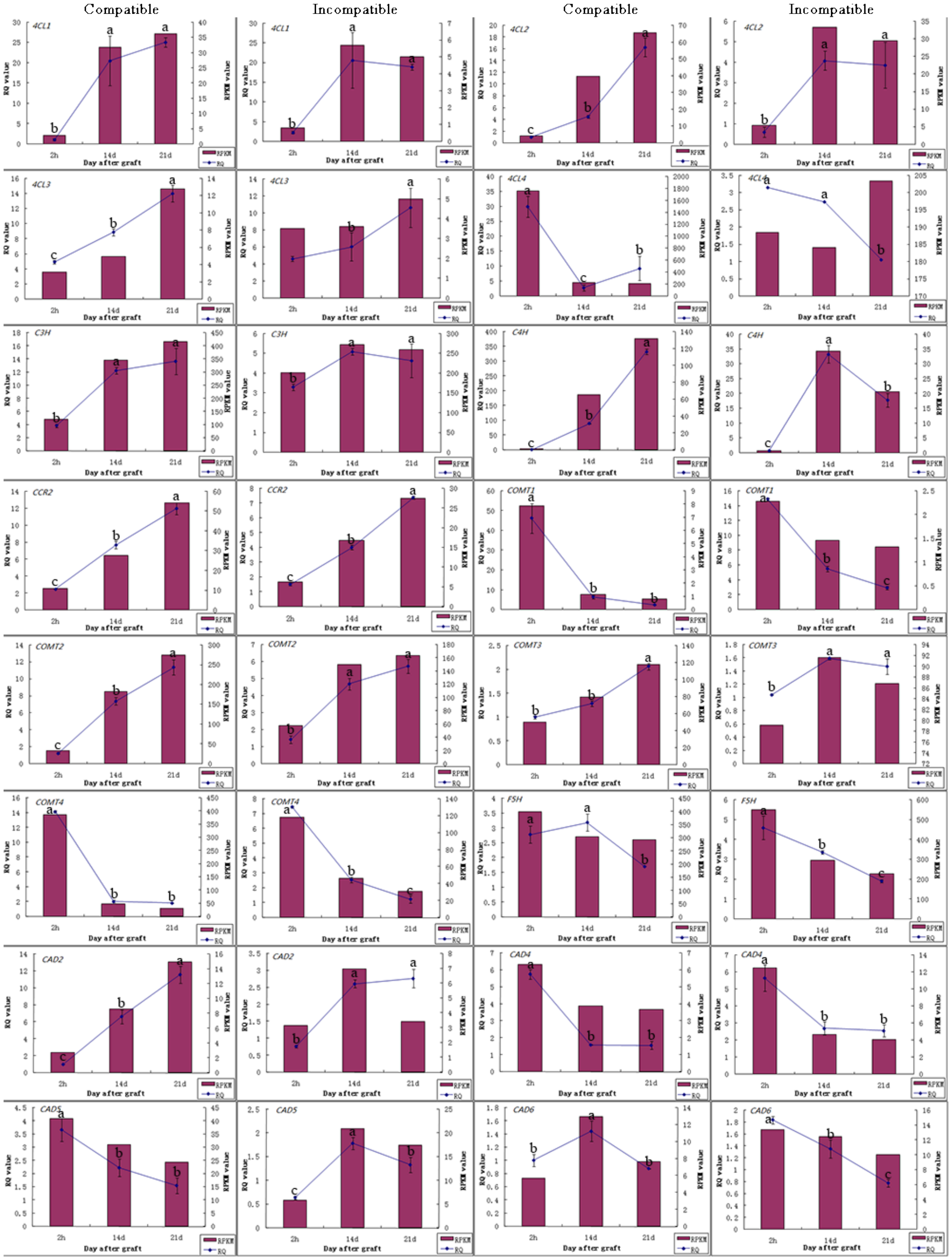
Compatible
Incompatible
Compatible
Incompatible
4CL1
4CL1
4CL2
4CL2
4CL3
4CL3
4CL4
4CL4
C3H
C3H
C4H
C4H
CCR2
CCR2
COMT1
COMT1
COMT2
COMT2
COMT3
COMT3
COMT4
COMT4
F5H
F5H
CAD2
CAD2
CAD4
CAD4
CAD5
CAD5
CAD6
CAD6
RQ value
RPKM value
Day after graft
2h
14d
21d
RPKM
RQ

Figure S7
